# Covid‐19 and Football: Crisis Creates Opportunity

**DOI:** 10.1111/1467-923X.12961

**Published:** 2021-02-03

**Authors:** Kieran Maguire

**Keywords:** football, Covid‐19, governance, US investment, ‘Big Six’

## Abstract

This article looks at the financial performance and position of English professional football before Covid‐19 and the impact that the pandemic has had on the industry. It analyses revenue streams in different divisions, the dependency that clubs have on them and how they have changed as a result of the pandemic. The article also reviews key costs for football clubs, the extent to which they can be reduced, different business models that operate, and possible funding sources for the sport from third parties and within the industry.

in 2020, covid‐19 reinforced the view that football is indeed the most important of the unimportant things in life. Somehow, the professional game managed to survive over the last calendar year, with the only notable casualty being Macclesfield Town, who were carefully stage‐managed out of existence as an English Football League (EFL) club.^1^ The administration of Wigan Athletic, and mysterious activities at Charlton, could not legitimately be levelled at the door of the virus, but at existing poor management and governance.^2^ The record of poor governance in football has attracted attention over many years, from parliamentary committees, individual MPs, supporters’ organisations and think tanks.

## Revenues

Football clubs generate revenue from three main revenue sources, plus the more volatile area of asset (player) sales, the latter providing the most column inches and speculation in print, broadcasting and social media outlets. Clubs across Europe have become reliant on player sales in the recent past, with some clubs making them a central part of their strategy, a reliance increased by the pandemic.

### Matchday

Matchday revenues (ticket sales) have been hit hardest by Covid‐19. The initial lockdown was followed by a pilot attempt to return fans to matches via Project Restart, which was subsequently reversed. Attempts to use behavioural carrots, in the form of allowing fans to return in very small numbers to parts of the country which were deemed to be tier 1 and 2, quickly evaporated. The new variant of Covid‐19 and subsequent lockdown signalled a return to matches taking place behind closed doors.

**Figure 1:**
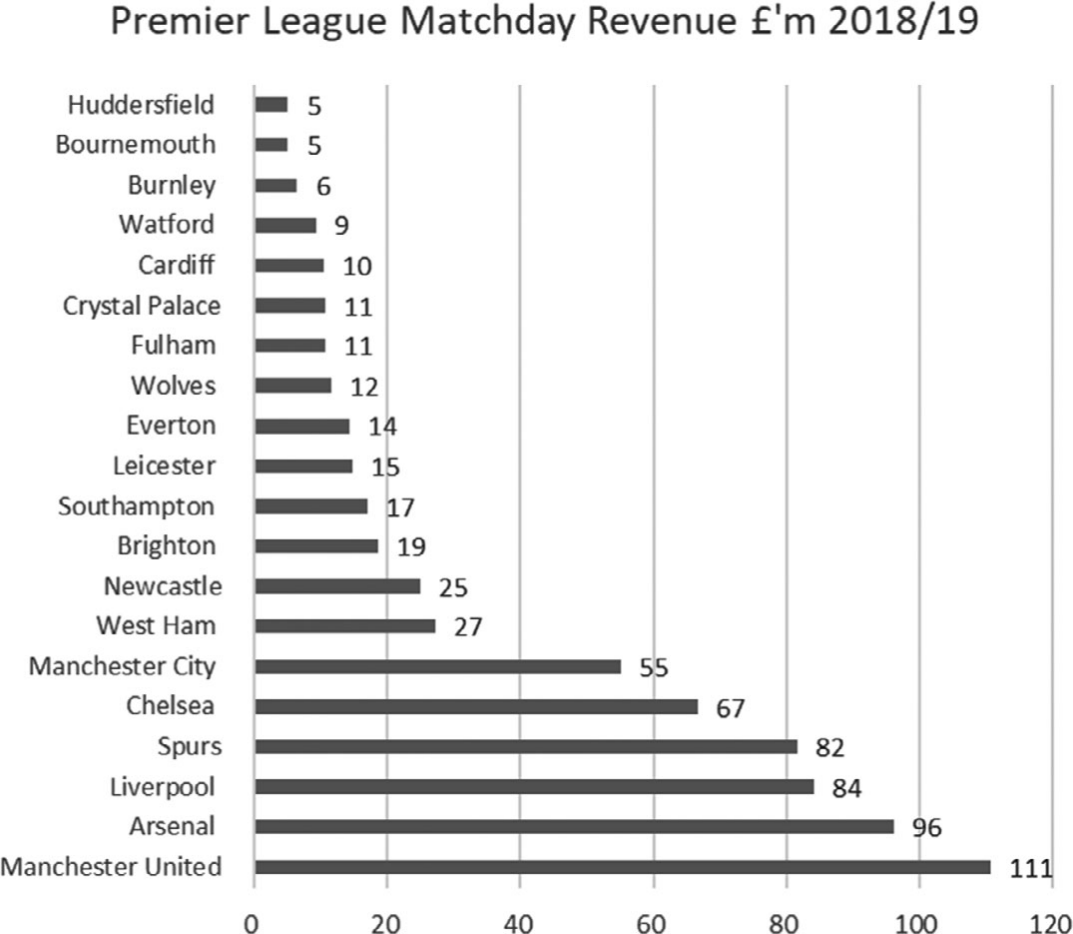
Premier League matchday revenue 2018/9

In the Premier League, matchday revenues accounted for 14 per cent of total income in 2018/19, this varies significantly from club to club. In total, matchday revenues generated £680 million for Premier League clubs in 2018/19. All matches were suspended on 13 March 2020 and when they returned, it was to empty stadiums. This has meant a reduction of about 20–25 per cent in matchday revenues for 2019/20 as clubs had to refund sums to season ticket holders and were unable to sell individual tickets. This will be amplified in 2020/21 owing to so few matches attracting paying fans.

For clubs in the EFL and below, there is greater reliance on matchday income and so these clubs have been hit relatively hard by having to play matches in empty stadiums. Championship clubs in receipt of parachute payments generated 13 per cent of revenue from matchdays in 2018/19, but for the remaining clubs, this was 28 per cent. The median matchday income for a Championship club for the 2018/19 season was £6 million.

In Leagues One and Two it is more difficult to get a true picture because so many clubs take advantage of Companies Act abbreviated accounts legislation and so produce accounts for small companies. This means they do not have to publish profit or loss accounts and the key data therein, such as revenues, operating profits and wages. However, from the limited data that is available, clubs in the bottom two divisions of the EFL generate 35–40 per cent of revenue from matchdays.

### Broadcast

The Premier League broadcast deal is the world’s most lucrative in the football industry. It generates just over £3 billion annually and is spread relatively democratically between clubs. In addition, four Premier League clubs qualify for the UEFA Champions League annually, and others for the UEFA Europa League. This can be worth up to €110 million in broadcast rights for a club that wins the Champions League.

Broadcasters’ reaction to the cancellation of matches has been mixed. Senior domestic broadcast partners such as Sky Sports and BT Sports are reliant upon a subscription model for their own income. Subscription holidays were initially given to customers during lockdown as the supply of live sports, not just football, dwindled during this period. There has been discussion with the Premier League in relation to providing an estimated rebate of £330 million owing to matches not taking place on contractually agreed dates during the lockdown commencing March 2020. To mitigate cash flow problems for clubs which were already short of matchday income, the domestic broadcasters appear to have agreed to spread the rebates due to them over a three‐year period. Increasing the number of matches made available to broadcasters, with no additional fees, also helped the relationship between the Premier League and its partners.

International broadcast deals have been more complicated. The Premier League’s three‐year deal with Chinese broadcaster PPTV, due to expire in 2022, was terminated after the broadcaster failed to make due payments. The PPTV deal was worth an estimated $700 million over the three‐year period. It has been replaced by a one‐year agreement with Tencent, but while the Premier League has not revealed the value, it is likely to be worth substantially less than the one it replaces.

Ten clubs in the Premier League are reliant on broadcast revenues for 75 per cent or more of total revenues. It was therefore imperative that matches took place in some shape or form, or higher rebates could have severely hit those clubs. The reliance upon broadcast income and fear of further rebates is a major factor in Premier League clubs wanting to fulfil fixtures in the 2020/21 season.

**Figure 2:**
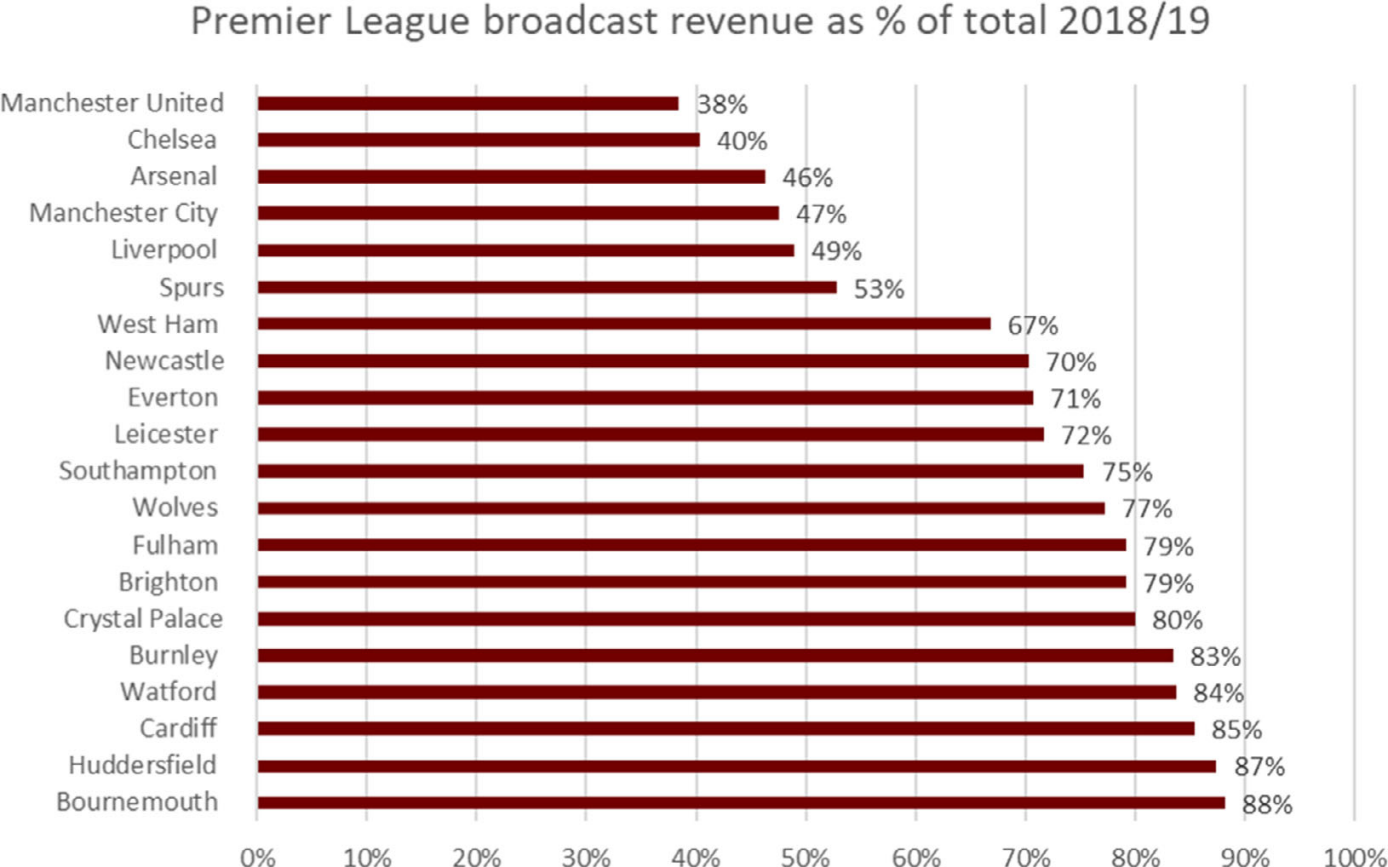
Premier League broadcast revenue 2018/9

Those clubs who participate in UEFA competitions will also be subject to a reduction in broadcast payments. This is partially because of matches not taking place on agreed dates, but also a reduction in the number of matches played. This is owing to UEFA making some knockout matches one‐legged instead of two.

The EFL broadcast deal is worth about £119 million a year, approximately 5 per cent of the value of the Premier League deal. The deal is broadly split 80 per cent to clubs in the Championship, 12 per cent to League One and 8 per cent to League Two. There has been a reported £7 million rebate agreed with Sky Sports as a result of Covid‐19 and lockdown.

There was an attempt to introduce pay‐per‐view (PPV) for Premier League matches in 2020/21. This resulted in a backlash from fan groups—not because of the principle, but the price point of £14.95 was deemed to be too high. However, PPV does exist in the EFL via the iFollow service and seems to be accepted by fans at £10 per match. Culturally, fans of Premier League clubs resented paying so much when, in their opinion, clubs were already generating large sums from existing revenue sources and many fans had subscriptions with broadcasters.

### Commercial

Most commercial deals are done on a club by club basis and individual financial deals tend to be scarce. Therefore, it is difficult to quantify the impact of Covid‐19 on this income stream. However, we have seen that West Ham’s sleeve sponsor, Basset & Gold, went into administration in April 2020, while Southampton’s front of shirt sponsor, LD Sports, abandoned the club in August 2020. Many clubs in the Premier League and Championship have sponsorships from betting companies—the extent of which has been controversial, but betting companies are fully aware that football audiences, both at matches and broadcast, are in line with their target demographic. Nevertheless, long‐term financial planning is also difficult: in the EFL many front of shirt sponsor deals were only agreed just before the 2020/21 season began.

## Costs

The most significant costs for football clubs relate to players—both wages and transfer fees.

### Wages

In the Premier League, wages have increased from £97 million in its first season, 1992/93, to £3,120 million in 2018/19. This is an increase of 2,811 per cent, compared to an increase in the RPI over the same period of 108 per cent.

Whilst it may appear that the increase in wages is unsustainable, football is a talent industry in which revenue drives costs, and the increase in revenue since the commencement of the Premier League in 1992 is broadly in line with wage increases. Although wages are often discussed and seem high, they are presently lower in the Premier League as a proportion of income than they were in 2003. The criticisms by senior politicians such as Matt Hancock of the wage levels being paid in the Premier League therefore seem misdirected.^3^ Football as an industry seems to be subject to a higher level of populist moral and financial scrutiny than almost any other.

**Figure 3:**
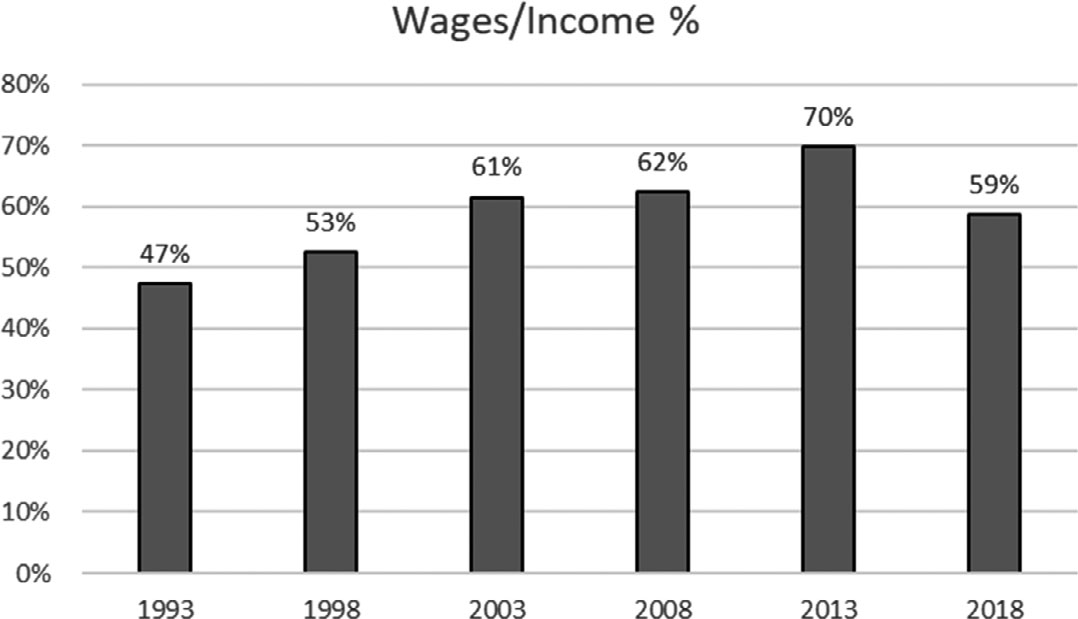
Wages as a percentage of Premier League income

There have been no wage cuts for players in the Premier League as a result of Covid‐19 except at Arsenal. Executives and managers at other Premier League clubs have taken pay cuts, and some players agreed to deferrals. Meanwhile, some Premier League clubs utilised the government’s Covid‐19 furlough scheme, resulting in hostility from fans. Liverpool, Spurs and Bournemouth all reversed utilisation of the furlough scheme following the negative response.^4^ This is further evidence of the different level of scrutiny and comment that appear to apply to football clubs when compared with similar sectors.

In the EFL, the position has been more challenging. Clubs competing in the Championship have for many years been running at a loss because their wages have exceeded revenues. Therefore, even pre Covid‐19 these clubs were very vulnerable to any form of economic shock. Covid‐19 is the most significant macroeconomic event in generations and as such has amplified the fiscally cavalier way in which clubs at this level have been managed.

**Figure 4:**
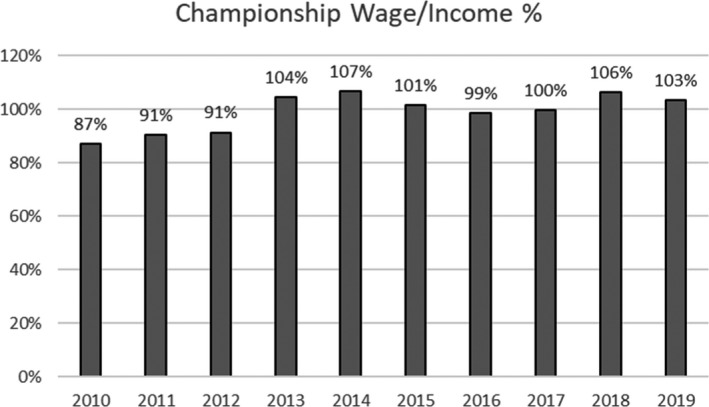
Wages as a percentage of income, Championship clubs

The response of Championship clubs to Covid‐19 has been mixed. Some have wealthy owners who have historically underwritten operating losses and they have continued to do so during the pandemic because they have the resources. For them, Covid‐19 has been an opportunity as much as a threat, as they can leverage on their financial advantages. Other clubs, with less wealthy owners, have been more enthusiastic to use the furlough scheme and arrange wage deferrals with players.

There has been no progress in applying any form of wage cap in the Championship, which would require a two‐thirds majority of clubs (sixteen votes) for any change in regulations. It is clear that there has not been enough support for any form of change from the existing ‘profitability and sustainability’ rules, introduced in 2011/12, which have had little impact on cost control, apart from providing much needed lucrative work for sports lawyers and accountants.

The reason clubs have been unable to force through pay cuts is that players have contractual wage agreements for fixed time periods. If the club breaches the terms, the player would in theory be in a position to walk away from the contract, and the club would not be entitled to compensation in the form of a transfer fee. The loss of transfer fee would often be greater than the cash saved by a wage cut. There would also be potential implications for staff (player) morale, which could impact upon results in the field of play. In the Premier League, each individual place in the table is calculated to be worth just under £2 million, so unhappy players who are not giving their full effort could result in significant sums of money being lost by clubs.

In Leagues One and Two, squad salary caps have been introduced for 2020/21, following Covid‐19, as an attempt to keep costs in check. In League One, the limit is £2.5 million and League Two, £1.5 million. There are caveats and clauses in relation to these salary caps that will make it challenging for the EFL to apply the rules. In some European countries and in Major League Soccer in the US there has been greater activity in terms of player wages cuts.

Covid‐19 has highlighted the gaps between individual divisions and the gambles that club owners have taken historically to bridge those gaps via promotion to a higher division. This could have been an opportunity to look at the cliff edges between divisions (the average revenue for a Premier League club is £258 million, compared to £32 million in the Championship, £6 million in League One and £4 million in League Two) and also within divisions themselves, where there is great inequality between those at the top and the bottom.

### Transfer fees

During the summer 2020 transfer window, Premier League clubs collectively spent an estimated £1,200 million on player recruitment.^5^ Culture Secretary Oliver Dowden questioned the wisdom of such spending when the UK government was considering financial support for sport. Whilst this is a valid observation, there appeared to be no appetite from Premier League clubs for financial support from central government during 2020 other than a desire to be able to generate revenue from having fans attend matches during periods when the level of infections was relatively low. Reductions in VAT from the present level of 20 per cent were mooted, but this is an irrelevance whilst matches are taking place in empty stadiums.

It should also be noted that if Premier League clubs spent £1,200 million in the transfer window, then some other clubs must be in receipt of the same amount of money. Significant sums were received by EFL clubs such as Bournemouth, Brentford, and Queens Park Rangers in selling their players to Premier League teams. Transfer spending in the other ‘Big Five’ leagues in Europe (Spain, Italy, Germany and France) fell significantly, reflecting the greater dependence in those countries on non‐broadcasting income. However, some Premier League clubs, such as Chelsea, have the benefit of wealthy owners such as Roman Abramovich, whose success and wealth in his other areas of business mean his motivations are not financial and he is in a position to bankroll spending at his club (which was approximately 20 per cent of the Premier League total in summer 2020).

In the EFL, by contrast, transfer spending was very muted, Championship clubs were only spending money if they had already successfully sold players for substantial fees. In the lower leagues nearly all transactions were loans or free transfers.

## Financial support

Central government has provided financial support to the football industry in the form of furlough and tax delay schemes. These have helped cash flow, especially in the early months of Covid‐19. The Premier League (via solidarity payments) and the EFL have advanced broadcast monies to EFL clubs that would usually be spread over the 2020/21 season. Although these are not additional funds, the timing is helpful, allowing clubs to meet their operational costs (especially wages) to date. The Premier League has also offered £50 million to clubs in Leagues One and Two, with extra funding in the form of grants which will help these clubs meet their obligations over the rest of the season. Whether this will prove to be sufficient is uncertain, given that there are additional Covid‐19 related costs, such as testing and transport (social distancing means that players cannot necessarily all travel in the same coach, for example). The Premier League has also offered a £200 million loan to Championship clubs to help them pay outstanding employment taxes owed to HMRC. However, this has created some resentment from those Championship clubs which had not fallen behind with their payments to government. Meanwhile, the National League has benefitted from central government support in the form of grants, but the distribution method used has resulted in controversy as some club owners felt they have been harshly treated.

In October 2020 Project Big Picture (PBP) was leaked to the press. This was an attempt by American club owners to take control of the English game. It was presented as a benevolent redistribution of money in the game from the Premier League to the EFL and a solution to the additional challenges arising from Covid‐19. A more detailed analysis revealed that PBP would have resulted in the gap in income in the Premier League between the ‘Big Six’ clubs (Liverpool, Manchester United, Manchester City, Spurs, Arsenal and Chelsea) and the remaining clubs, which presently averages £350 million a year, would increase. Furthermore, only six votes would have been required to change policies in the Premier League and other aspects of the domestic game, allowing operational and strategic control of professional football to be concentrated in the hands of a few owners, whose motives might be self serving rather than for the whole industry.

## Summary

Covid‐19 has highlighted the existing financial and governance weaknesses in domestic football and these are likely to receive continuing attention from policy makers. The industry has done well so far to survive the loss of revenue and disruption of the sport with relatively few casualties. Like many parts of the service/entertainment sector of the economy, football is in a precarious position and desperate for a successful vaccination programme to allow a return of spectators to reduce the financial losses incurred to date. Even so, if there is a return to fans attending matches this does not address the structural and systematic issues that presently exist in the industry.

## Notes

^1^
J. Freeman, ‘Macclesfield Town FC wound up in High Court over debts exceeding £500,000’, BBC Sport, 16 September 2020; https://www.bbc.co.uk/sport/football/54177582 (accessed 13 January 2021).

^2^
D. Conn, ‘Brutal and bizarre: the story of how Wigan collapsed into administration’, *The Guardian*, 2 July 2020; https://www.theguardian.com/football/2020/jul/02/story‐of‐how‐wigan‐collapsed‐into‐administration‐au‐yeung‐investigation; T. Doyle, ‘Charlton Athletic confirm Thomas Sandgaard is the club’s new owner’, *Evening Standard*, 25 September 2020; https://www.standard.co.uk/sport/football/charlton‐athletic‐fc‐news‐new‐owner‐thomas‐sandgaard‐confirmed‐a4556336.html (both accessed 13 January 2021).

^3^
‘Coronavirus: Premier League players should take a pay cut—Matt Hancock’, BBC Sport, 2 April 2020; https://www.bbc.co.uk/sport/football/52142267 (accessed 13 January 2021).

^4^
O. Dornay, ‘Bournemouth become latest Premier League club to reverse decision to furlough staff after “listening to supporters”’, talkSPORT, 14 April 2020; https://talksport.com/football/694648/bournemouth‐furlough‐staff‐coronavirus/ (accessed 13 January 2021).

^5^
D. Roan, ‘Premier League and EFL “close” over bailout, says Culture Secretary Oliver Dowden’, BBC Sport, 19 November 2020; https://www.bbc.co.uk/sport/football/55008860 (accessed 13 January 2021).

